# Protective Effect of *Akkermansia muciniphila* against Immune-Mediated Liver Injury in a Mouse Model

**DOI:** 10.3389/fmicb.2017.01804

**Published:** 2017-09-26

**Authors:** Wenrui Wu, Longxian Lv, Ding Shi, Jianzhong Ye, Daiqiong Fang, Feifei Guo, Yating Li, Xingkang He, Lanjuan Li

**Affiliations:** ^1^State Key Laboratory for Diagnosis and Treatment of Infectious Diseases, The First Affiliated Hospital, School of Medicine, Zhejiang University, Hangzhou, China; ^2^Collaborative Innovation Center for Diagnosis and Treatment of Infectious Diseases, Hangzhou, China; ^3^Department of Gastroenterology, Sir Run Run Shaw Hospital, Zhejiang University Medical School, Hangzhou, China

**Keywords:** *Akkermansia muciniphila*, liver injury, acute hepatitis, microbiota, immune regulation

## Abstract

Accumulating evidence indicates that gut microbiota participates in the pathogenesis and progression of liver diseases. The severity of immune-mediated liver injury is associated with different microbial communities. *Akkermansia muciniphila* can regulate immunologic and metabolic functions. However, little is known about its effects on gut microbiota structure and function. This study investigated the effect of *A. muciniphila* on immune-mediated liver injury and potential underlying mechanisms. Twenty-two C57BL/6 mice were assigned to three groups (*N* = 7–8 per group) and continuously administrated *A. muciniphila* Muc^T^ or PBS by oral gavage for 14 days. Mouse feces were collected for gut microbiota analysis on the 15th day, and acute liver injury was induced by Concanavalin A (Con A, 15 mg/kg) injection through the tail vein. Samples (blood, liver, ileum, colon) were assessed for liver injury, systemic inflammation, and intestinal barrier function. We found that oral administration of *A. muciniphila* decreased serum ALT and AST and alleviated liver histopathological damage induced by Con A. Serum levels of pro-inflammatory cytokines and chemokines (IL-2, IFN-γ, IL-12p40, MCP-1, MIP-1a, MIP-1b) were substantially attenuated. *A. muciniphila* significantly decreased hepatocellular apoptosis; *Bcl-2* expression increased, but *Fas* and *DR5* decreased. Further investigation showed that *A. muciniphila* enhanced expression of *Occludin* and *Tjp-1* and inhibited *CB1* receptor, which strengthened intestinal barriers and reduced systemic LPS level. Fecal 16S rRNA sequence analysis indicated that *A. muciniphila* increased microbial richness and diversity. The community structure of the Akk group clustered distinctly from that of mice pretreated with PBS. Relative abundance of Firmicutes increased, and Bacteroidetes abundance decreased. Correlation analysis showed that injury-related factors (IL-12p40, IFN-γ, *DR5*) were negatively associated with specific genera (*Ruminococcaceae_UCG_009*, *Lachnospiraceae_UCG_001*, *Akkermansia*), which were enriched in mice pretreated with *A. muciniphila*. Our results suggested that *A. muciniphila* Muc^T^ had beneficial effects on immune-mediated liver injury by alleviating inflammation and hepatocellular death. These effects may be driven by the protective profile of the intestinal community induced by the bacteria. The results provide a new perspective on the immune function of gut microbiota in host diseases.

## Introduction

Hepatitis and its complications are still a worldwide health burden. Numerous environmental insults can cause hepatic damage, and viral infections and autoimmune activation are major causative factors. Hepatitis pathogenesis is associated with T cell stimulation and immunologic injury (Li et al., 2016a).

Emerging evidence has shown a strong linkage between intestinal flora and liver diseases (Tilg et al., 2016). Different microbial communities may participate in the onset and progression of liver diseases, with strikingly varied microbial compositions reported (Lozupone et al., 2012; Celaj et al., 2014; Llopis et al., 2016). These findings suggest that modulating the composition and function of intestinal flora may be a potential intervention for liver injury. However, studies on the relationship between microbiota and immune-mediated liver injury are rare.

Concanavalin A (Con A) injection is used to establish a liver injury model resembling autoimmune liver diseases and virus hepatitis, which is characterized by T cell-mediated hepatocellular death and inflammatory infiltration (Heymann et al., 2015). Multiple cytokines, including IFN-γ, TNF-α, IL-12, and IL-2, are involved in the pathogenesis of this fulminant liver injury. A recent study found that gut microbiota is involved in the pathogenesis of the Con A model, as differential susceptibility to Con A challenge was observed in mice from different vendors (Celaj et al., 2014). Furthermore, Chen et al. (2014) reported that intragastric administration of pathogenic bacteria aggravated Con A-induced liver injury. However, it is unclear whether increasing functional autochthonous microbes can change liver susceptibility and improve liver injury.

*Akkermansia muciniphila* is a Gram-negative anaerobic bacterium and the only isolated representative of the Verrucomicrobia phylum (Derrien et al., 2004). This resident constitutes at least 1% of the total fecal bacteria among humans and is also detected in other mammals (Belzer and de Vos, 2012). Researchers showed that *A. muciniphila* can degrade mucin, a primary component of the mucus layer, which enables close contact of the bacteria with intestinal cells under the mucus (Derrien et al., 2011). Mucin degradation also provides nutrition for intestinal commensals and produces short chain fatty acids (SCFAs) (Belzer and de Vos, 2012). Colonization of germ-free (GF) mice with *A. muciniphila* altered expression of mucus genes related to immune response and homeostasis (Derrien et al., 2011).

Recent studies have shown that the abundance of *A. muciniphila* is inversely correlated with several diseases, including inflammatory bowel disease, appendicitis, obesity, and autism (Zhang et al., 2009; Png et al., 2010; Wang et al., 2011; Dao et al., 2016). Moreover, *A. muciniphila* treatment can attenuate atherosclerosis and protect against high fat diet-induced metabolic disorders in animal models (Everard et al., 2013; Li et al., 2016b; Plovier et al., 2017). These findings suggest that this indigenous resident has a role in host immune regulation and metabolic function due to the beneficial microbial composition promoted by this bacterium. However, the exact effect of *A. muciniphila* on intestinal microbial communities is rare. This study aimed to investigate the effect of *A. muciniphila* on Con A-induced liver injury, as well as the underlying mechanisms.

## Materials and Methods

### Strains and Growth Conditions

*Akkermansia muciniphila* Muc^T^ was grown anaerobically in a basal mucin-based medium at 37°C (Derrien et al., 2004). The bacteria were incubated under an atmosphere of 10% H_2_, 10% CO_2_, and 80% N_2_ (AW300SG anaerobic workstations; Electrotek, England). The cultures were centrifuged (6000 rpm, 10 min, 4°C), washed twice with sterile anaerobic PBS, and resuspended at a final concentration of 1.5^∗^10^10^ CFU/mL under strictly anaerobic conditions. For analysis of the viability of *A. muciniphila*, the suspension was inoculated on a mucin-based medium containing 1% agarose and then incubated for at least 1 week at 37°C in the anaerobic incubator.

### Mice and Treatment

**FIGURE 1:**
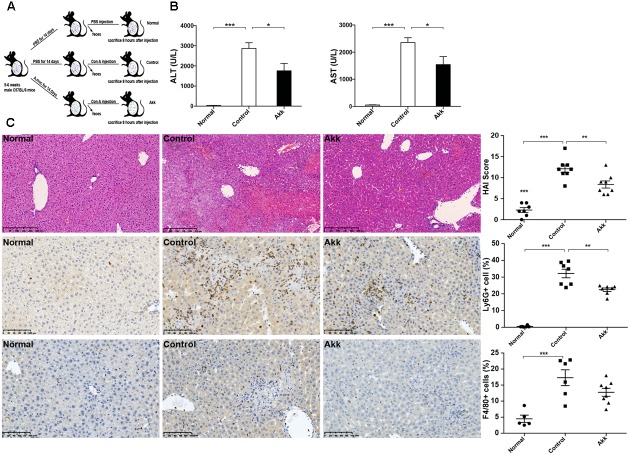
*Akkermansia muciniphila* administration inhibited Con A-induced acute liver injury. **(A)** Design of the animal experiment. Mice were randomly distributed to three groups (Normal, *n* = 8; Control, *n* = 7; Akk, *n* = 7). Mice were pretreated with PBS or *A. muciniphila* continuously for 14 days by gavage. On day 15, feces from each mouse were collected, and Con A was injected. Eight hours later, mice were sacrificed. **(B)** Serum levels of ALT and AST (*n* = 7–8 per group). **(C)** Representative liver histology. Upper panel: Left, H&E staining, scale bar, 250 μm; Right, modified HAI scores of liver histopathology. Middle panel: Left, staining of neutrophils (Ly6G+), scale bar, 100 μm; Right, percentage of Ly6G+ cells. Lower panel: Left, staining of macrophages (F4/80+), scale bar, 100 μm; Right, percentage of F4/80+ cells. Data are shown as the mean ± SEM. ^∗^*P* < 0.05; ^∗∗^*P* < 0.01; ^∗∗∗^*P* < 0.001 by *post hoc* ANOVA one-way statistical analysis.

SPF male C57BL/6 mice (4–5 weeks) were purchased from Shanghai SLAC Laboratory Animal, Co., Ltd. All animals were maintained under specific pathogen-free conditions. After 1–2 weeks of acclimation, mice were randomly assigned to three groups. Group Akk (*A. muciniphila*+ Con A) mice were administrated 3^∗^10^9^ CFU *A. muciniphila* suspended in 0.2 ml sterile anaerobic PBS by oral gavage per day, while mice in Group Control (PBS + Con A) and Group Normal (PBS + PBS) were given an equivalent volume of sterile anaerobic PBS instead. Treatment was continued for 14 days (**Figure 1A**). All mice were maintained in a temperature- and humidity-controlled environment under a 12-h light–dark cycle and had *ad libitum* access to food and water. All experiments were approved by the Local Committee of Animal Use & Protection and were performed in accordance with criteria of “Guide for the Care and Use of Laboratory Animals” (NIH publication 86–23 revised 1985).

### Model of Acute Liver Injury

Concanavalin A (15 mg/kg, Sigma–Aldrich, St. Louis, MO, United States) was dissolved in pyrogen-free PBS and applied to mice in the Akk and Control groups through intravenous injection via tail veins. Mice from Group Normal were injected with PBS as healthy controls. Mouse feces (0.18–0.22 g) were collected before Con A injection. Mice were sacrificed 8 h after the Con A challenge. Blood, tissue (liver, ileum, colon) and cecal content were collected.

### Transaminase Activity and Endotoxin Assay

Serum alanine aminotransferase (ALT) and aspartate transaminase (AST) levels were measured to evaluate liver function using a standard clinical automated analyzer (SRL, Tokyo, Japan). Plasma concentration of LPS was determined using a kinetic turbidimetric Endotoxin assay kit (Chinese Horseshoe Crab Reagent Manufactory, Co., Ltd., Xiamen, China).

### Analysis of Histopathology and Immunohistochemistry

Liver specimens were immediately fixed in 10% neutral buffered formalin after collection. Four-μm-thick paraffin-embedded tissues were stained with hematoxylin and eosin (H&E). The modified histologic activity index (HAI) was applied to assess liver inflammation and hepatocyte death in a blinded manner. Hepatocyte apoptosis was detected by TUNEL assays with an In Situ Cell Death Detection Kit, POD1 kit (Roche, United Kingdom) according to the instructions. Proximal colon tissues were incubated in Carnoy’s solution after removal, and paraffin sections were used for Alcian blue staining. For immunohistochemistry, the liver sections were stained with F4/80 (macrophages) and Ly6G (neutrophils). Positive cells were countered using Image J software.

### Measurement of Serum Cytokines and Chemokines

Serum cytokine concentration was determined by a 23-plex assay kit (Bio-Plex Pro Mouse Cytokine 23-Plex Panel, Bio-Rad, Hercules, CA, United States) and analyzed using MAGPIX system (Luminex Corporation) and Bio-Plex Manager 6.1 software (Bio-Rad) according to the manufacturer’s instructions. The kit assessed the following cytokines and chemokines: IL-1a, IL-1b, IL-2, IL-3, IL-4, IL-5, IL-6, IL-9, IL-10, IL-12(p40), IL-12(p70), IL-13, IL-17, Eotaxin, G-CSF, GM-CSF, IFN-γ, KC, MCP-1, MIP-1a, MIP-1b, RANTES, and TNF-α.

### Bacterial Translocation Assay

Mesenteric lymph nodes (MLNs), renal samples, and a piece of the left liver lobe were aseptically collected and weighed in autoclaved glass homogenizers, separately. After the samples were thoroughly homogenized with sterile saline (doses adjusted according to weight), tissue homogenates were diluted and plated in duplicate on brain heart infusion agar (BHI, Oxoid, Thermo Fisher Biochemicals, Ltd., Beijing, China). Plates were separately incubated under anaerobic and aerobic conditions at 37°C for 48 h. An equivalent volume of sterile saline was plated on BHI agar as a control. Number of mice was recorded if the culture was positive.

### RNA Extraction and Real-time Quantitative PCR Analysis (qPCR)

Tissue samples were collected at sacrifice in tubes containing Allprotect Tissue Reagent (Qiagen, Hilden, Germany) and stored at -80°C. Total RNA was extracted from the liver and ileum (15–20 mg) using an RNeasy Mini Kit (Qiagen, Hilden, Germany), following the manufacturer’s protocols. The mRNA expression was measured in duplicate with a One Step SYBR PrimeScript plus RT-PCR Kit (TaKaRa Biotechnology, Co., Ltd., Dalian) using a 7500 real-time PCR system (Applied Biosystems). The relative mRNA expression of target genes was assessed using the comparative cycle threshold (Ct) method and normalized to β-actin expression. Primer pairs are listed in Supplementary Table 1.

### Metagenome Sequencing of the 16S rRNA Gene

Feces were immediately incubated on ice and rapidly transferred to -80°C for storage after collection. Bacterial DNA was extracted using a QIAamp Fast DNA Stool Mini Kit (Qiagen, Hilden, Germany) according to manufacturer’s instructions. PCR primers targeting the V3–V4 region of the 16S rRNA gene with specific barcodes were used (338F: 5′-ACTCCTACGGGAGGCAGCA-3′, 806R: 5′-GGACTACHVGGGTWTCTAAT-3′). After amplification, sequencing was performed using Illumina MiSeq.

### Bioinformatics Analysis

Sequencing reads were merged, filtered, and clustered to operational taxonomical units (OTUs) according to the 97% sequence identity. Taxonomic analysis was conducted by the RDP classifier using representative OTUs, based on the Silva Database^1^. The community richness (Chao1 and Ace) and diversity (Shannon and Simpson) were analyzed by Mothur software (v.1.30.1) at 97% identity. Principal co-ordinate analysis (PCoA) was conducted using R software based on Weighted UniFrac distances. Linear discriminant analysis (LDA) coupled with effect size measurement (LefSe) analysis was performed online^2^. Spearman’s rho non-parametric correlation was adopted to analyze relationships between microbiota and liver injury-related indexes, and the results were graphically visualized using Cytoscape (Shannon et al., 2003).

### Statistical Analysis

An unpaired *t*-test or Wilcoxon rank-sum test was used to analyze differences between two groups. One-way analysis of variance (ANOVA) was performed for significance analysis among groups. All statistical analyses were performed using SPSS 20.0 (SPSS, Inc., Chicago, IL, United States) with *P* < 0.05 considered statistically significant. Figures were generated by Prism 6.0 (GraphPad Software, Inc.).

## Results

### Pretreatment with *A. muciniphila* Improved Con A-Induced Liver Injury

The efficiency of intragastric infusion was verified by the higher relative abundance of *Akkermanisa* in both the feces and cecum compared to that of the controls (Supplementary Figure 1). *Akkermanisa* abundance was previously reported to be associated with reduced body weight (Everard et al., 2013; Plovier et al., 2017). This was also observed in our study, as mice treated with *A. muciniphila* presented the lowest body weight among the groups (Supplementary Figure 1).

After gavage administration, acute autoimmune liver injury was established by Con A injection, as shown by elevated serum ALT and AST (**Figure 1B**). Compared to the positive control (Control group, administered with PBS), *A. muciniphila* treatment significantly reduced the Con A-induced elevation of serum ALT and AST activities. H&E liver staining showed that Con A injection induced substantial congestion and portal inflammation, and massive hepatocyte death was detected in the periportal and intralobular areas. However, there was only sporadic degeneration, and the inflammatory infiltration was much milder in the Akk group compared to that of the Control group. Besides, immunohistochemistry staining showed a markedly decreased infiltration of neutrophils (Ly6G+) and a lower trend of macrophages (F4/80+) in group Akk (**Figure 1C**). The significantly decreased score by modified HAI in the Akk group indicated reduced liver damage (**Figure 1C**).

### *A. muciniphila* Alleviated Con A-Induced Immune Reactions by Modulating Cytokine Secretion

**FIGURE 2:**
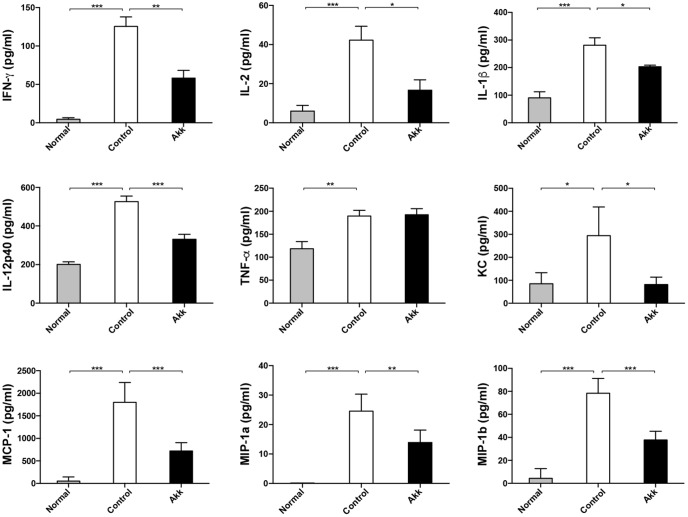
Pretreatment with *A. muciniphila* relieved Con A-induced cytokine expression in the serum. The increased production of IFN-γ, IL-2, IL-1β, and IL-12p40 was significantly diminished, along with reduced chemokines, including KC, MCP-1, MIP-1a, and MIP-1b. However, TNF-α concentration was not evidently reduced. Data are shown as the mean ± SEM. *N* = 5–8 per group. ^∗^*P* < 0.05, ^∗∗^*P* < 0.01, ^∗∗∗^*P* < 0.001 by *post hoc* ANOVA one-way statistical analysis or Kruskal–Wallis test.

Con A-induced hepatic damage is predominantly mediated through a broad range of pro-inflammatory cytokines, especially IFN-γ, TNF-α, and IL-2. To determine whether *A. muciniphila* administration could ameliorate the production of inflammation factors, we assessed profiles of serum cytokines. Eight hours after Con A challenge, concentrations of pro-inflammatory cytokines, including IFN-γ and IL-2, dramatically increased, accompanied by high levels of chemokines (MCP-1, MIP-1a, MIP-1b, and KC) (**Figure 2**, Control vs. Normal). In contrast, *A. muciniphila* significantly reduced the increase in IFN-γ, IL-2, IL-1β, and IL-12p40, although the concentration of TNF-α, another crucial factor, was unchanged. In addition, levels of the chemokines mentioned were substantially reduced.

### *A. muciniphila* Suppressed Con A-Induced Hepatocyte Apoptosis

**FIGURE 3:**
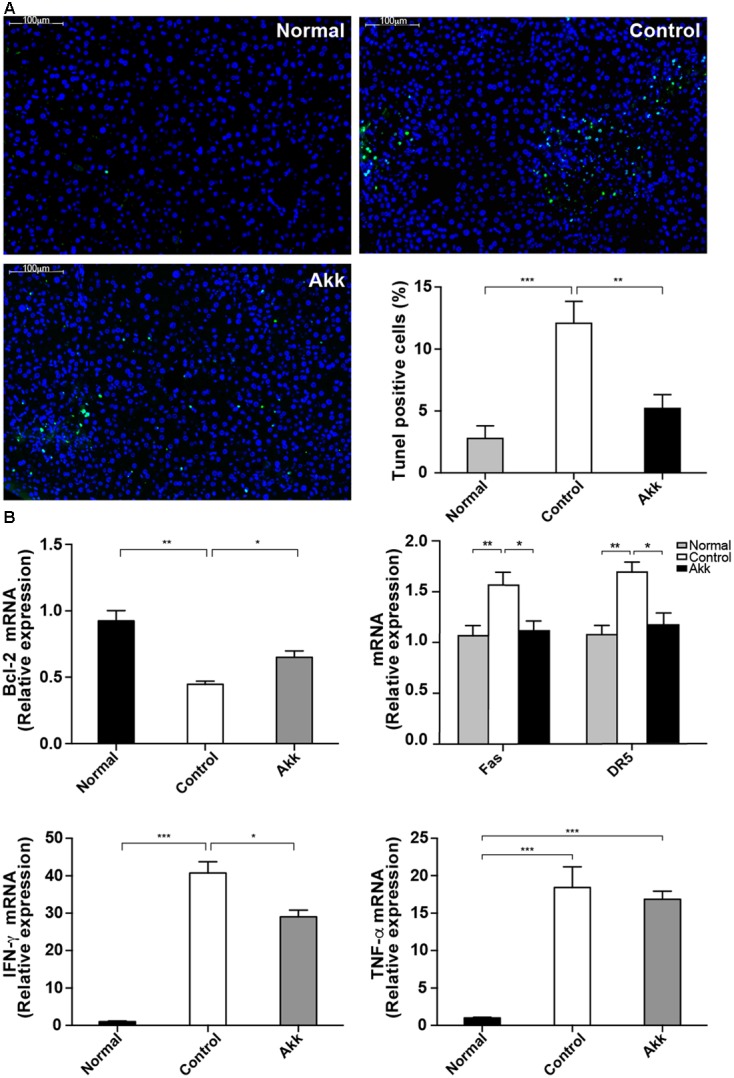
*Akkermansia muciniphila* suppressed Con A-induced hepatocyte apoptosis. **(A)** TUNEL assays of liver histology 8 h after Con A injection. There was extensive damage in group Control, but only sporadic apoptosis was observed in group Akk (scale bar, 100 μm). Percentage of apoptotic cells in the liver was significantly lower in group Akk (*n* = 7–8 per group). **(B)** Liver expression of genes related to apoptosis and inflammation (*n* = 6–8 per group). mRNA level of an important anti-apoptotic factor, *Bcl-2*, was markedly reduced in group Control, whereas it was significantly elevated in group Akk. Expression of both *Fas* and *DR5* was diminished. *IFN-*γ was substantially reduced in group Akk, but expression of *TNF-*α was not significantly influenced. Data are shown as the mean ± SEM. ^∗^*P* < 0.05, ^∗∗^*P* < 0.01, ^∗∗∗^*P* < 0.001 by *post hoc* ANOVA one-way statistical analysis.

To explore the potential mechanism underlying the protective effects of *A. muciniphila* on hepatic cells, we next assessed hepatocellular apoptosis. After treatment with *A. muciniphila*, the strong plant lectin only resulted in sporadic apoptotic cells, while more extensive apoptosis was observed in the Control group (**Figure 3A**). Additionally, the percentage of TUNEL-positive cells prominently dropped to 5.2% due to the pretreatment compared to 12.1% in the Control group.

Furthermore, we detected significantly enhanced expression of *Bcl-2* mRNA in liver tissue from the *A. muciniphila*-pretreated mice (**Figure 3B**), and *Bcl-2* is considered a major anti-apoptosis factor. Cytotoxic factors, including FasL/Fas and TNF-related apoptosis-inducing ligand (TRAIL)/DR5, have been proposed to participate in T-cell-induced hepatocyte death (Song et al., 2003; Zheng et al., 2004). Therefore, we measured *Fas* and *DR5* levels in the liver. Both were significantly inhibited in mice pretreated with *A. muciniphila* (*Fas*/β*-actin r*atios: 1.04 ± 0.09 vs. 1.46 ± 0.12, *P* < 0.05; *DR5*/β*-actin* ratios: 1.09 ± 0.11 vs. 1.57 ± 0.09, *P* < 0.05, compared to Controls). Consistent with the serum results, *IFN-*γ expression in the liver was substantially reduced in group Akk, while *TNF-*α expression was not significantly influenced (**Figure 3B**).

### *A. muciniphila* Reinforced Gut Barrier Function

To characterize the gut barrier function, we examined the differences in bacterial translocation and systemic endotoxin between the Control and Akk groups. The translocation percentages of anaerobes were 57.1% vs. 85.7% (4/7 vs. 6/7, Akk vs. Control) in MLNs and 0% vs. 42.9% (0/7 vs. 3/7, Akk vs. Control) in both liver and renal samples, although this difference was not significant due to the sample size (**Table 1**). Similar trends were found for aerobic translocation (71.4% vs. 100% in MLNs, 57.1% vs. 71.4% in liver, 14.3% vs. 71.4% in renal, Akk vs. Control). Accordingly, plasma concentration of LPS was markedly decreased in group Akk compared to group Control (**Table 1**), providing extra evidence of a strengthened barrier function after *A. muciniphila* supplement.

**Table 1:** Bacterial translocation and systemic endotoxin among groups.

	Normal	Control	Akk	*P*^∗^
**MLN %(n/N)**				
Anaerobe	12.5 (1/8)	85.7 (6/7)	57.1 (4/7)	0.237
Aerobe	12.5 (1/8)	100 (7/7)	71.4 (5/7)	0.070
**Liver %(n/N)**				
Anaerobe	0 (0/8)	42.9 (3/7)	0 (0/7)	0.257
Aerobe	12.5 (1/8)	71.4 (5/7)	57.1 (4/7)	0.577
**Renal %(n/N)**				
Anaerobe	0 (0/8)	42.9 (3/7)	0 (0/7)	0.051
Aerobe	0 (0/8)	71.4 (5/7)	14.3 (1/7)	0.031
LPS (EU/ml)^#^	/	0.85 ± 0.28	0.12 ± 0.08	0.032

^∗^*Comparison between group Control and Akk by Chi-square test for categorical data and Mann–Whitney test for LPS; ^#^LPS in group Normal was under the detectable line (0.007 EU/ml)*.

**FIGURE 4:**
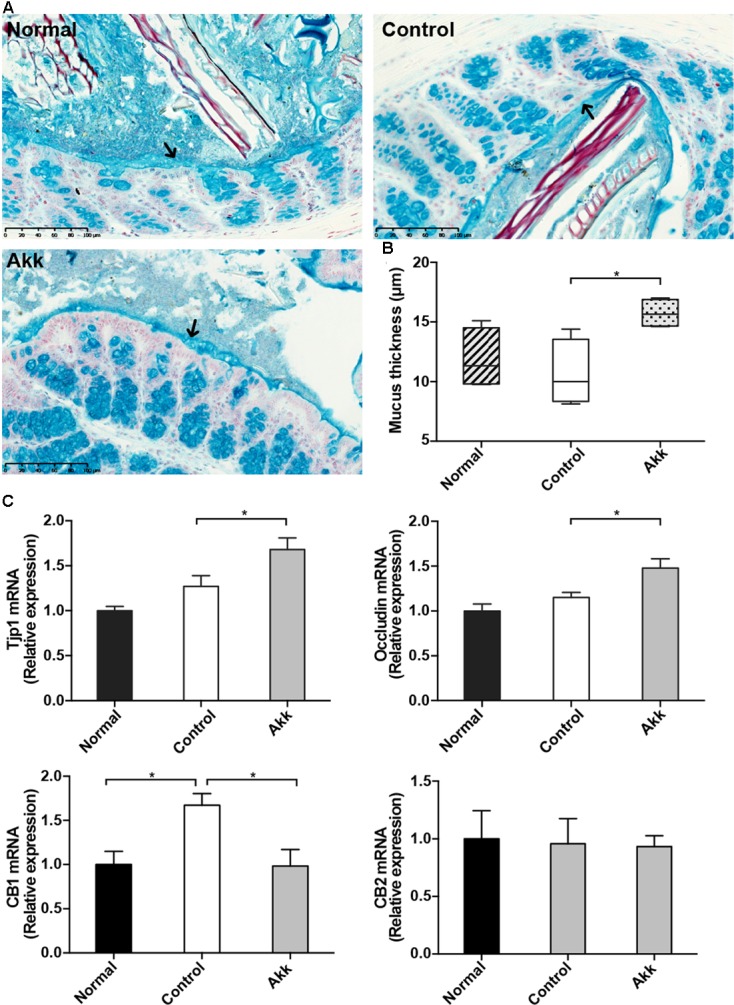
*Akkermansia muciniphila* reinforced gut barrier function. **(A)** Representative Alcian blue images of the inner mucus layer. Scale bar, 100 μm. **(B)** Thickness measurement of the inner mucus layer by Alcian blue staining. Data are expressed as Min to Max. **(C)** mRNA expression of *Tjp1*, *Occludin*, *CB1*, and *CB2* in the ileum. Data are shown as the mean ± SEM. ^∗^*P* < 0.05 by *post hoc* ANOVA one-way statistical analysis.

Previous studies showed that colonization of *A. muciniphila* aids in the refreshment and reshaping of the mucus layer, and mucus production was proposed to be involved in the colonization resistance (Belzer and de Vos, 2012). We examined the thickness of the inner mucus layer covering the proximal colon by Alcian blue staining. There was no significant reduction of mucus thickness between groups Control and Normal, which suggested Con A challenge did not have a significant effect on the mucus production. Strikingly, this protective layer evidently thickened in the Akk group compared with that of the Control group, indicating that pretreatment strengthened the intestinal barrier (**Figures 4A,B**). We next evaluated the intestinal expression of *Tjp1* (which encodes zonula occludens 1) and *Occludin* in the ileum. *Tjp1* transcription was upregulated by 32%, and *Occludin* was increased by 28% after administration of *A. muciniphila* compared with that of the Control group (**Figure 4C**). It was reported that Cannabinoid Receptor 1 (CB1) participates in control of gut permeability and is positively related with LPS levels (Muccioli et al., 2010; Plovier et al., 2017). We found expression of *CB1* was significantly increased after Con A injection, while in *A. muciniphila* pretreated-mice it remained similar to Normal group. And *CB2* was not altered among groups (**Figure 4C**). However, there was no significantly different expression of antimicrobial peptides such as *Lyz1*, *DefA*, and *Reg3g* between group Akk and Control, with the exception that *Pla2g2* was much lower in group Akk (Supplementary Figure 2).

### *A. muciniphila* Increased Microbial Diversity and Reshaped the Microbial Community

**FIGURE 5:**
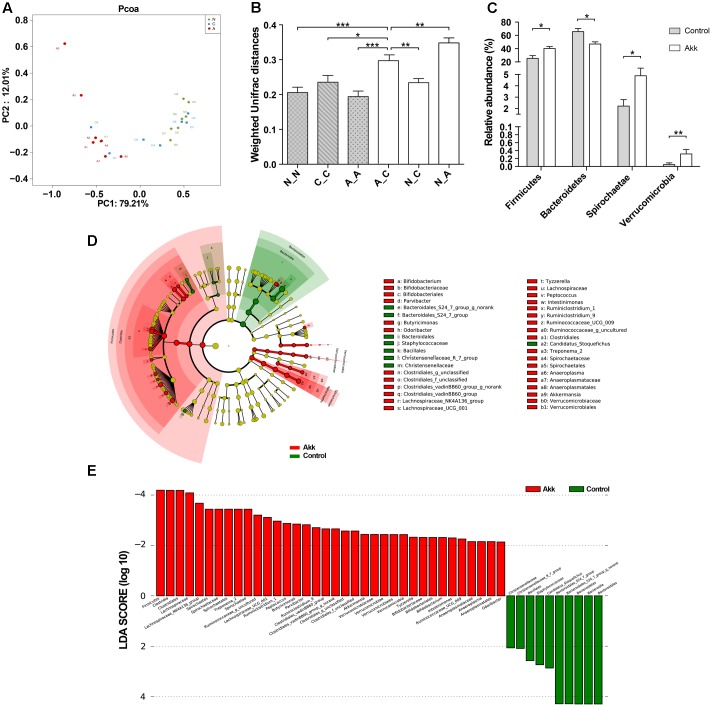
Alteration of the microbial community. **(A)** PCoA among three groups based on Weighted UniFrac distances. Each point represented a sample. N, group Normal; C, group Control; A, group Akk. **(B)** Comparisons of pair-wised Weighted Unifrac distances. ANOVA was conducted for multiple comparisons with the LSD method for correction. N_N, C_C, and A_A, indicate intragroup distances; A_C, N_C, and N_A, indicate intergroup distances. **(C)** Relative abundance of taxa at the phylum level in group Control and Akk. Significance was determined by Wilcoxon rank sum tests. **(D)** LEfSe cladograms represented taxa enriched in group Control (green) and group Akk (red). Rings from the inside out represented taxonomic levels from phylum to genus. Sizes of circles indicate relative abundance of the taxon. **(E)** Discriminative biomarkers with twofold changes in LDA scores. Data are shown as the mean ± SEM. ^∗^*P* < 0.05, ^∗∗^*P* < 0.01, ^∗∗∗^*P* < 0.001.

To investigate the influence of *A. muciniphila* intake on gut microbiota composition, we assessed fecal pellets by 16S rRNA sequencing. In contrast to the Control group, *A. muciniphila* administration significantly improved commensal richness and diversity as shown by consistently elevated OTUs, Ace, and Shannon indexes in the Akk group (**Table 2**). In contrast, the Simpson index was lower in the group Akk, which also indicated a higher microbial diversity. However, these indexes were unchanged between Groups Normal and Control, both of which were only administered PBS. We next examined altered microbial construction by PCoA clustering using weighted UniFrac distance (β diversity). Commensal composition of the Akk group clustered distinctly from that of the other two groups on PC1, which explained 79.21% of the variance (**Figure 5A**). Additionally, overall commensal structures of the Normal and Control groups behaved similarly since they received the same treatment although they were raised in separate cages. We further evaluated pairwise variation between samples based on their cages and pretreatments by weighted Unifrac distances (**Figure 5B**). Intergroup β diversity (N_A or A_C) was significantly greater than any intragroup distance (C_C, N_N, and A_A), while pairwise distance between the Normal and Control groups (N_C) was relatively equivalent to intragroup ones. This indicated a distinct role of *A. muciniphila* in modulating microbial structures.

**Table 2:** Richness and diversity of fecal microbiota among groups.

Index	Normal	Control	Akk
OTUs	439 ± 42	429 ± 48	485 ± 19^∗^
Ace	496 ± 43	481 ± 40	535 ± 22^∗^
Chao1	499 ± 39	491 ± 35	537 ± 23
Shannon index	4.137 ± 0.242	4.193 ± 0.289	4.635 ± 0.178^∗,#^
Simpson index	0.039 ± 0.012	0.039 ± 0.014	0.021 ± 0.005^∗,#^

^∗^*Compared to group Control; ^#^Compared to group Normal; *post hoc* ANOVA one-way statistical analysis; OTUs, operational taxonomic units*.

### *A. muciniphila* Administration Altered Relative Abundance of Taxa at Multiple Levels

To further characterize phenotypic changes in the taxonomic composition, we compared relative taxa abundance at the phylum and genus levels using Wilcoxon rank sum tests. The two dominant phyla in mouse feces were Bacteroidetes and Firmicutes. After continuous administration of *A. muciniphila*, Firmicutes abundance substantially increased (40% in group Akk, 25% in group Control, *P* < 0.05), and the Bacteroidetes proportion decreased (47% in group Akk, 66% in group Control, *P* < 0.05). Additionally, abundance of Spirochaetae and Verrucomicrobia was also significantly elevated due to the treatment (**Figure 5C**). Analysis of the taxonomic alteration showed that taxa between the Normal and Control groups shared comparable features at the genus level, while relative abundance of particular genera in group Akk was dramatically different compared to that of the Control (Supplementary Figure 3).

We then performed LEfSe analysis to identify differentially abundant biomarkers with biological consistency between the Akk and Control groups (Segata et al., 2011). Bacteroidetes and Firmicutes were separately enriched in group Control and group Akk. Similarly, we also found that bacteria-treated mice harbored a distinctively higher abundance of the phylum Verrucomicrobia, which was due to *Akkermansia*, compared to that of the untreated mice. Other taxa specifically enriched were also observed between the two groups at multiple phylogenetic levels (**Figure 5D**). According to Wilcoxon analysis and the LDA score (**Figure 5E**), families such as *Lachnospiraceae* (containing genera *Lachnospiraceae_UCG-001* and *Lachnospiraceae_NK4A*) and *Spirochaetaceae* (predominantly due to genus *Treponema_2*) were significantly associated with group Akk. Although abundance of the family *Ruminococcaceae* was comparable between the two groups, several genera within the family tended to be enriched in group Akk, including *Ruminococcaceae_g_uncultured*, *Ruminococcaceae_UCG_009*, and *Ruminiclostridium_9*. Furthermore, *Staphylococcaceae* and *Bacteroidales_S24_7_group* were relatively deficient in group Akk compared to that of group Control.

### *A. muciniphila*-Modified Gut Microbiota Influenced Host Response to Con A Challenge

**FIGURE 6:**
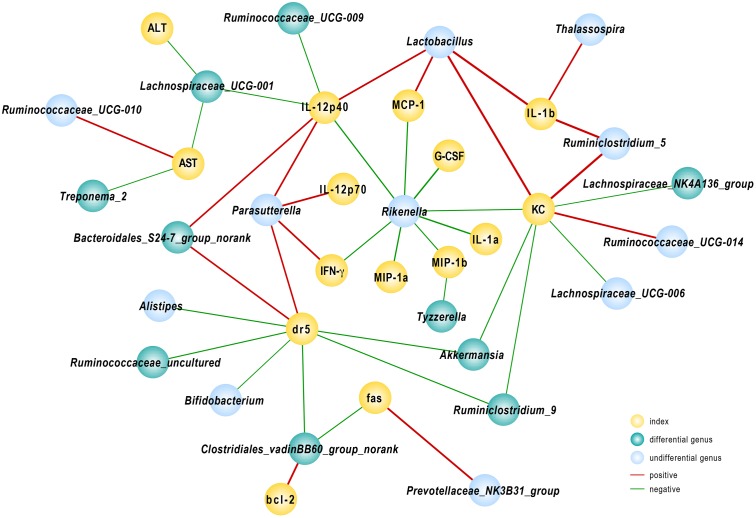
Correlation analysis between gut microbiota and injury-related indexes. Spearman’s rho non-parametric correlation was used, and significant relationships with *P* < 0.05 and *r* > 0.5 are shown. Yellow nodes: injury-related indexes. Green nodes: differentially distributed genera between Control and Akk groups; blue nodes: the indexes showed differences but were not significantly different between the groups. Red lines between nodes represent positive relationships, and blue ones indicate negative linkages. The thickness of the connection represents the correlation coefficient, with thicker lines indicating higher *r* values.

To further elucidate the consequences of altered community construction, we performed correlation analysis between the relative abundance of bacteria at the genus level and injury parameters. Several cytokines were associated with gut microbiota. As one of the primary pro-inflammatory cytokines, IL-12(p40) was negatively related to population densities of the genera *Ruminococcaceae_UCG-009* and *Lachnospiraceae_UCG-001* and positively associated with *Lactobacillus* and *Bacteroidales_S24-7_group_norank*. The *Lachnospiraceae_UCG-001* population, prominently abundant in group Akk, likewise reflected the severity of liver damage as indicated by ALT and AST levels, as *r* = -0.57 and -0.54, respectively (*P* < 0.05). We found that the fecal population of the genus *Rikenella* had a significant negative relationship with almost all tested pro-inflammatory cytokines and chemokines. Among the relevant inflammatory factors, IL-1α and G-CSF had the strongest negative correlations, with Spearman rank coefficients of -0.85 and -0.87, respectively (*P* < 0.05). The *DR5* gene, evidently down-regulated due to *A. muciniphila* as shown above, had a significantly negative association with *Akkermansia*, *Ruminiclostridium_9*, *Bifidobacterium*, and *Clostridiales_vadinBB60_group_norank* and a positive correlation with abundance of *Bacteroidales_S24-7_group_norank*. Several genera listed above were substantially enhanced in *A. muciniphila*-treated mice (**Figure 6**). These data provided evidence that modified gut microbiota influenced the host response to Con A challenge.

## Discussion

Accumulating evidence has indicated the beneficial roles of *A. muciniphila* in metabolic and immunologic disorders (Shin et al., 2014; Plovier et al., 2017). This bacterium can improve glucose tolerance and ameliorate metabolic system inflammation and is considered a promising probiotic (Everard et al., 2013; Shin et al., 2014; Plovier et al., 2017). However, few studies have focused on its microbial modulation. Additionally, whether *A. muciniphila* also has a favorable role in immune-related liver injury remains unknown. Our study investigated the specific effects of *A. muciniphila* on Con A-induced liver damage and elucidated the potential mechanisms. We found that *A. muciniphila* pretreatment alleviated liver damage by altering transaminase activities and histologic injuries. Systemic inflammation was attenuated as cytokines, including IFN-γ, IL-1β, IL-2, and IL-12p40, were suppressed. The beneficial effects of *A. muciniphila* were partially dependent on strengthened gut barrier and the altered composition and function of gut flora. This study demonstrated that intestinal microbiota participated in the susceptibility of T cell-mediated liver injury, and *A. muciniphila* could reshape the intestinal microbial community to a beneficial and more protective profile.

Con A can activate T cells to attack hepatocytes, which resembles autoimmune liver disease and virus hepatitis (Schumann and Tiegs, 1999). Recent research found that gut microbiota prominently influence the population and function of DCs and NKT cells in the Con A model (Chen et al., 2014). In addition to the infiltration of effector cells, multiple cytokines, including IFN-γ, TNF-α, IL-12, and IL-2, are responsible for liver damage (Wang et al., 2012). In our research, serum levels of IFN-γ in the Akk group were prominently diminished, accompanied by lower IL-2, IL-12p40, and IL-1β. There was a significant reduction in many chemokines, such as MCP-1, MIP-1a, MIP-1b, and KC. These chemokines variously attract neutrophils, lymphocytes, and macrophages to the liver, accelerating disease progression. The immunohistochemical staining proved decreased infiltration of neutrophils and macrophages due to lower chemokines in the Akk group.

As previously reported, apoptosis is prevalent in acute liver damage (Li et al., 2016a). Fas/FasL and TRAIL/ TRAIL receptor 2 (DR5) have been shown to participate in the pathogenesis of Con A-induced liver injury. The liver constituently expresses Fas and TRAIL receptors/DRs (only DR5 in mice) (Zheng et al., 2004), each containing a death domain whose activation can induce apoptosis (Schumann and Tiegs, 1999). Blockage of either molecule can significantly ameliorate hepatocyte death and inflammation (Seino et al., 1997; Zheng et al., 2004). Our experiments showed significantly decreased expression of *Fas* and *DR5*, suggesting that *A. muciniphila* pretreatment regulates expression of death-related receptors following challenge by Con A. Furthermore, a negative relationship between *Akkermansia* abundance and *DR5* mRNA expression was also observed. Previous studies revealed that *A. muciniphila* is reduced in obese children and high-fat diet-fed mice (Karlsson et al., 2012; Everard et al., 2013). *A. muciniphila* influences metabolism of fatty acids and bile acids (Lukovac et al., 2014). DR5 was found to be up-regulated in NAFLD patient livers and was also associated with free fatty acids and bile acids. Moreover, *in vitro* experiment further identified detrimental roles of DR5 in hepatocyte apoptosis (Malhi et al., 2007). These studies showed the relationships between gut bacteria and *DR5* expression, which was confirmed in our experiment. Additionally, there was a significantly higher mRNA level of *IFN-*γ in livers from the group Control compared to that of group Akk. Elevated IFN-γ can bind to IFNGR and inhibit transcription of *Bcl-2*; *Bcl-2* is considered to be a potent anti-apoptosis factor and has beneficial effects on Con A-activated hepatocyte death (Li et al., 2016a).

Multiple studies have examined the increased intestinal permeability and bacterial translocation in chronic liver diseases (Bajaj et al., 2014; Tilg et al., 2016; Ubeda et al., 2016), while its role in T cell-mediated liver injury is unclear. It has been reported that LPS level is markedly elevated after Con A injection, which suggests an impaired gut barrier (Lin et al., 2012). And gut-derived pathogenic bacteria aggravates liver injury caused by Con A, which can be reversed by antibiotic decontamination (Lin et al., 2012; Chen et al., 2014). Our experiment detected lower plasma levels of LPS and decreasing trends of bacteria translocation to MLNs, liver, and renal tissues, which was related to the reinforced gut barrier induced by *A. muciniphila* via a thickened mucus layer, enhanced tight junctions and lower expression of CB1.

As the primary barrier of the colon, the mucus layer overlying the intestinal epithelium defends against external disturbances, including pathogen invasion and toxins. Thinner gel layers will increase gut permeability and increase the risk of bacterial translocation and endotoxemia. Mucus is produced by goblet cells and is refreshed every hour (Johansson et al., 2013); its production relies on the colonization of commensal bacteria or exposure to microbial toxins, whereas germ-free mice have extremely in colonic mucus which can be quickly restored by bacteria or their products (Petersson et al., 2011). Reunanen et al. (2015) also discovered that *A. muciniphila* can fortify enterocyte integrity *in vitro*. *In vivo*, *A. muciniphila* can promote the replenishment of mucus, improve the impaired intestinal barrier and thus relieve systemic inflammation (Everard et al., 2013). Previous studies found that Amuc_1100, one of the membrane proteins from *A. muciniphila* can specifically activate Toll-like receptor (TLR) 2, which can modulate the epithelial barrier function (Cario et al., 2007; Plovier et al., 2017). Ottman et al. (2017) also found that *A. muciniphila* induces pro- and anti-cytokines production of peripheral blood mononuclear cells. This indicated a complicated role of *A. muciniphila* in modulation of intestine homeostasis.

It was reported that endocannabinoid (eCB) system plays a role in gut homeostasis including motility, inflammation and permeability, of which the underlying mechanisms remains to be elucidated (Lee et al., 2016; Karwad et al., 2017). CB1 blockage was shown to improve gut barrier function and reduce systemic LPS levels, while the other member of eCB system-CB2 seemed unrelated with permeability (Muccioli et al., 2010). The gut microbiota was considered to regulate gastrointestinal function through eCB system (Lee et al., 2016). Everard et al. (2013) also found that *A. muciniphila* supplement elevates endocannabinoid content. Our experiment consistently found that Con A injection markedly up-regulated CB1 expression with elevated plasma LPS levels and administration of *A. muciniphila* was able to reverse this alteration. Whether the beneficial effect was attributed to the altered intestinal microbiota or *A. muciniphila* itself needs further investigation.

Gut flora have been shown to participate in the regulation of hepatocellular response to Con A and alcohol (Celaj et al., 2014; Llopis et al., 2016) since reconstitution of GF mice results in more severe liver damage, and mice fed separated showed inconsistent responses to lectin (Celaj et al., 2014). The explanation may partially lie in different constituents and dynamics of intestinal flora. A previous study showed that mice raise separately harbor unique microbiota (Celaj et al., 2014). Consistent with these findings, we also found slightly distinct structures in the microbial communities between separated cages (group Con and Normal). However, the microbiota of *A. muciniphila*-treated mice was distantly clustered from those of these two groups, providing support for the important role of *A. muciniphila* in reshaping intestinal community. Several studies have investigated the modulating effect of *A. muciniphila* on commensal flora with different doses and times of induration in mice (Everard et al., 2013; Org et al., 2015). Everard et al. (2013) found no significant alteration of gut microbiota after 4 weeks administration of 10^8^ CFU per day, whereas the construction of microbiota has changed following 5 weeks’ administration of 10^9^ CFU dosage (Org et al., 2015). In our experiment with 2 weeks’ administration of 3^∗^10^9^ CFU per day, we observed a structural disparity in gut microbiota. The different dosages may partially explain the discrepancy, since a lower dose (4.0^∗^10^6^ CFU) of *A. muciniphila* is unable to ameliorate glucose tolerance (Shin et al., 2014). This implied that higher dosage was needed to exert regulating impacts on resident gut microbiota. Besides, other variations such as different samples (feces or cecum) and methodologies (Mouse Intestinal Tract Chip or 16S rRNA gene sequence) may also be involved.

We observed *A. muciniphila* supplement predominantly increased Firmicutes, including the families *Lachnospiraceae* and *Ruminococcaceae*. *Lachnospiraceae* and *Ruminococcaceae* are considered beneficial autochthonous taxa, which are less abundant in acute-on-chronic liver failure (ACLF) and cirrhosis and are negatively associated with disease severity (Bajaj et al., 2014; Chen et al., 2015). Both *Lachnospiraceae* and *Ruminococcaceae* are well-known for their butyrate-producing properties, and butyrate can elicit anti-inflammatory effects. We also observed that some genera within these families had negative associations with pro-inflammatory cytokines and apoptotic factors, indicating favorable roles of these microbes for health. *Rikenellaceae* is a common colonizer in the intestine and is negatively associated with cirrhosis severity (Bajaj et al., 2014). Our study showed that *Rikenella* was linked to almost all tested cytokines, and *Alistipes* was correlated with *DR5* expression. Furthermore, both of the two genera belong to *Rikenellaceae* were enriched in group Akk, although not significantly. This provided extra support for the beneficial role of microflora induced by *A. muciniphila*.

*A. muciniphila* is the predominant mucolytic bacteria in healthy subjects and is considered a marker of a healthy gut (Belzer and de Vos, 2012). Other infrequent mucolytic bacteria, such *Ruminococcus gnavus* and *Ruminococcus torques*, may disproportionately increase when illness occurs and are positively related to disease severity (Prindiville et al., 2004; Png et al., 2010; Chen et al., 2014). The distribution of *A. muciniphila* is positively correlated with *Ruminococcaceae*, which was enriched in *A. muciniphila*-treated mice in our study (Arumugam et al., 2011). *A. muciniphila* is capable of utilizing mucin, which in turn produces oligosaccharides and SCFAs. *A. muciniphila* and its metabolites have a strong effect on gene transcription, and most genes are associated with metabolism and cell death receptor signaling (Derrien et al., 2011; Lukovac et al., 2014). The metabolites are also sources of energy for some gut commensals, which may in turn alter the relative abundance of SCFA-dependent microbes and regulate commensal interactions (Belzer and de Vos, 2012). SCFAs have been widely shown to have anti-inflammatory effects on innate immune cells and intestinal epithelial cells (Fusunyan et al., 1999; Ciarlo et al., 2016). These data indicate that the beneficial effects of *A. muciniphila* were mediated not just by the bacteria *per se* but also by the protective bacterial profile it induced, as confirmed by the increased community richness and diversity and microbial correlation with various parameters.

There are some limitations in our study. It remains unclear whether the differential expression of genes already existed after gavage or occurred after injection, and further studies are needed to elucidate the host response to *A. muciniphila* treatment in healthy mice. While significant biomarkers were identified, associated taxa at the species level were not investigated due to the sequence length. Although we observed distinct distributions of microbiota and several linkages between bacteria and injury-related parameters, there may still exist potential mechanisms that need to be fully elucidated.

## Conclusion

Our findings demonstrated that gut microbiota could influence the susceptibility to and severity of acute liver injury induced by Con A in mice. *A. muciniphila* increased the diversity and richness of the microbial community and simultaneously modulated the varieties of gut microbes, such *Lachnospiraceae* and *Ruminococcaceae*. The reshaped microbiota was correlated with reduced inflammatory cytokines and cytotoxic factors. These findings provide a new perspective on relationships between microflora and the host, suggesting *A. muciniphila* as a promising probiotic with beneficial effects on liver diseases.

## Author Contributions

WW, LjL, and LxL designed the study; WW and DS performed the experiments; JY, DF, and FG analyzed the data; WW, XH, and YL drafted the manuscript. All authors contributed to and approved the final article.

## Conflict of Interest Statement

The authors declare that the research was conducted in the absence of any commercial or financial relationships that could be construed as a potential conflict of interest.

## Footnotes

1 http://rdp.cme.msu.edu/

2 http://huttenhower.sph.harvard.edu/galaxy/
