# Supplementary material for: Protective Effect of Akkermansia muciniphila against Immune-Mediated Liver Injury in a Mouse Model
Source: Front Microbiol. 2017 Sep 26;8:1804. doi: 10.3389/fmicb.2017.01804 (PMC5626943; doi:10.3389/fmicb.2017.01804)
Supplement: Supplementary file 1 [file Data_Sheet_1.DOCX]

Supplementary Material

Protective Effect of *Akkermansia muciniphila* against Immune-mediated Liver Injury in a Mouse Model

**Wenrui Wu^1,2^, Longxian Lv^1,2^, Ding Shi^1,2^, Jianzhong Ye^1,2^, Daiqiong Fang^1,2^, Feifei Guo^1,2^, Yating Li^1,2^, Xingkang He^3^, Lanjuan Li^1,2^***

^1^State Key Laboratory for Diagnosis and Treatment of Infectious Diseases, The First Affiliated Hospital, School of Medicine, Zhejiang University, Hangzhou, China

^2^Collaborative Innovation Center for Diagnosis and Treatment of Infectious Diseases, Hangzhou, China

^3^Department of Gastroenterology, Sir Run Run Shaw Hospital, Zhejiang University Medical School, Hangzhou 310016, China

*** Correspondence:** Lanjuan Li: [ljli@zju.edu.cn](mailto:ljli@zju.edu.cn)

# Supplementary Figures and Tables

## Supplementary Figures

**Supplementary Fig 1. Relative abundance of Akkermansia in intestinal flora and mouse body weight.** (A) Population of the genus Akkermansia in feces after 14 days of gavage administration and in cecum content 8 hours after Con A injection. (B) Mouse body weight after 14 days of gavage administration. Data are shown as the mean ± SEM (n = 6-8 per group). *, P < 0.05; **, P < 0.01 by post hoc ANOVA one-way statistical analysis or Wilcoxon rank-sum test.

**
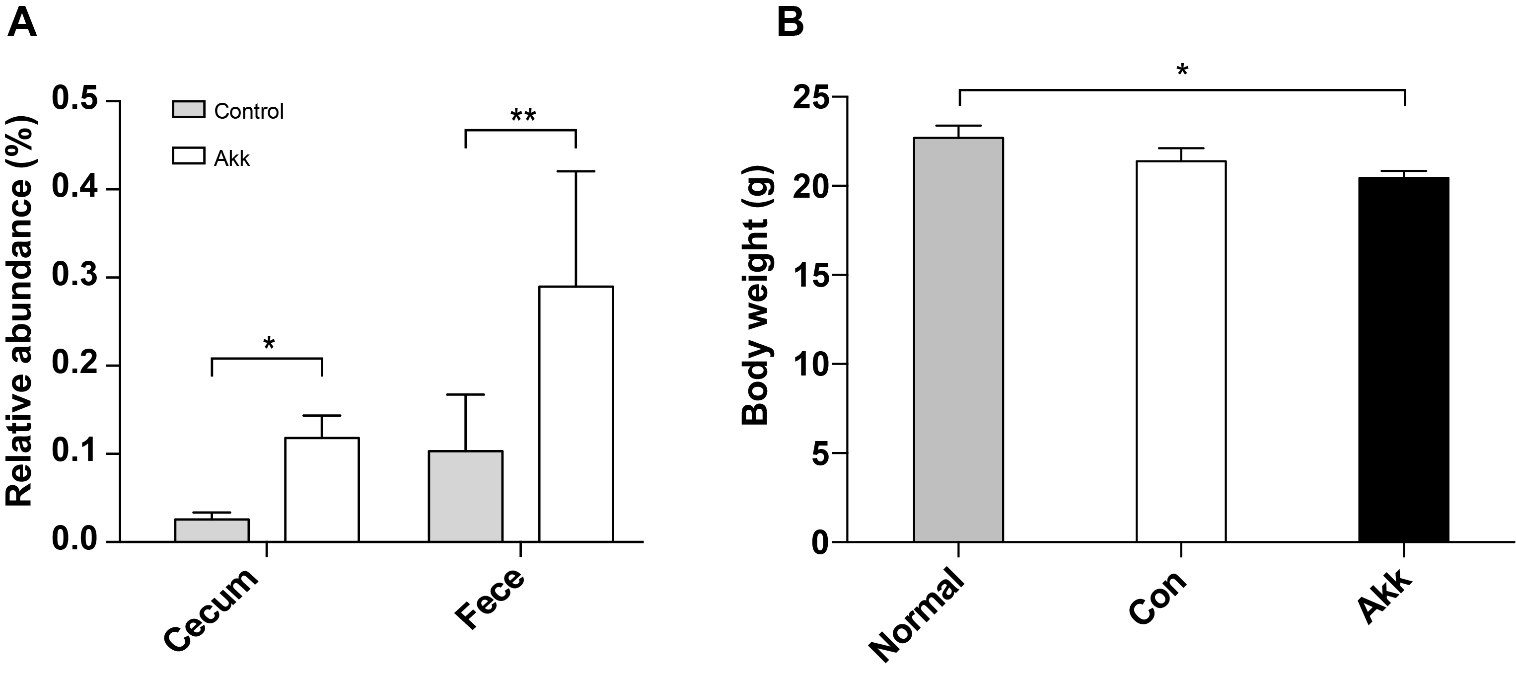
**

**Supplementary Fig 2. Relative expression of antimicrobial peptides in ileum.** Data are shown as the mean ± SEM. *, P < 0.05 by post hoc ANOVA one-way statistical analysis.

**
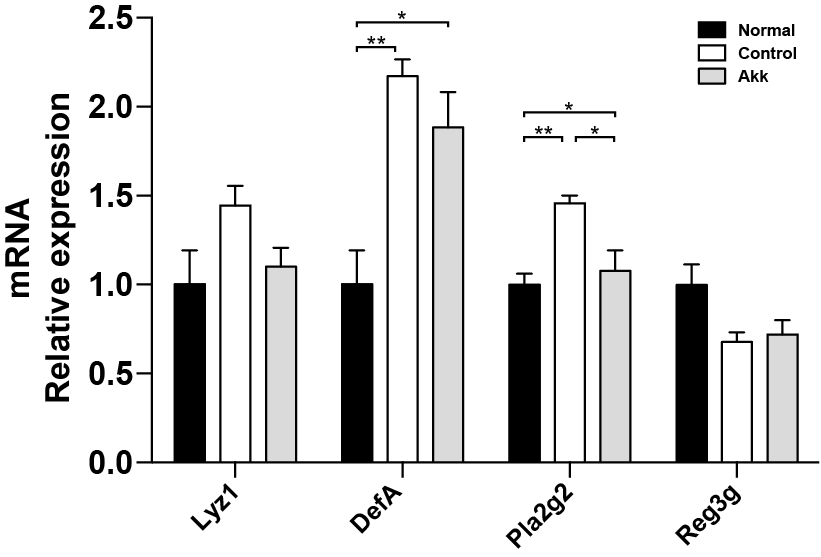
**

**Supplementary Fig 3. Taxonomic alteration of fecal microbiota in mice from different groups at the genus level.** (A) General characterization of the predominant genera (relative abundance >1%) among groups. Each column represents a single sample (n = 7-8 per group). N, group Normal; C, group Control; A, group Akk. (B) Significantly differentiated genera between the Control and Akk groups are shown, with P < 0.05 by Wilcoxon rank-sum test. Data are shown as the mean ± SEM (n = 7 per group).


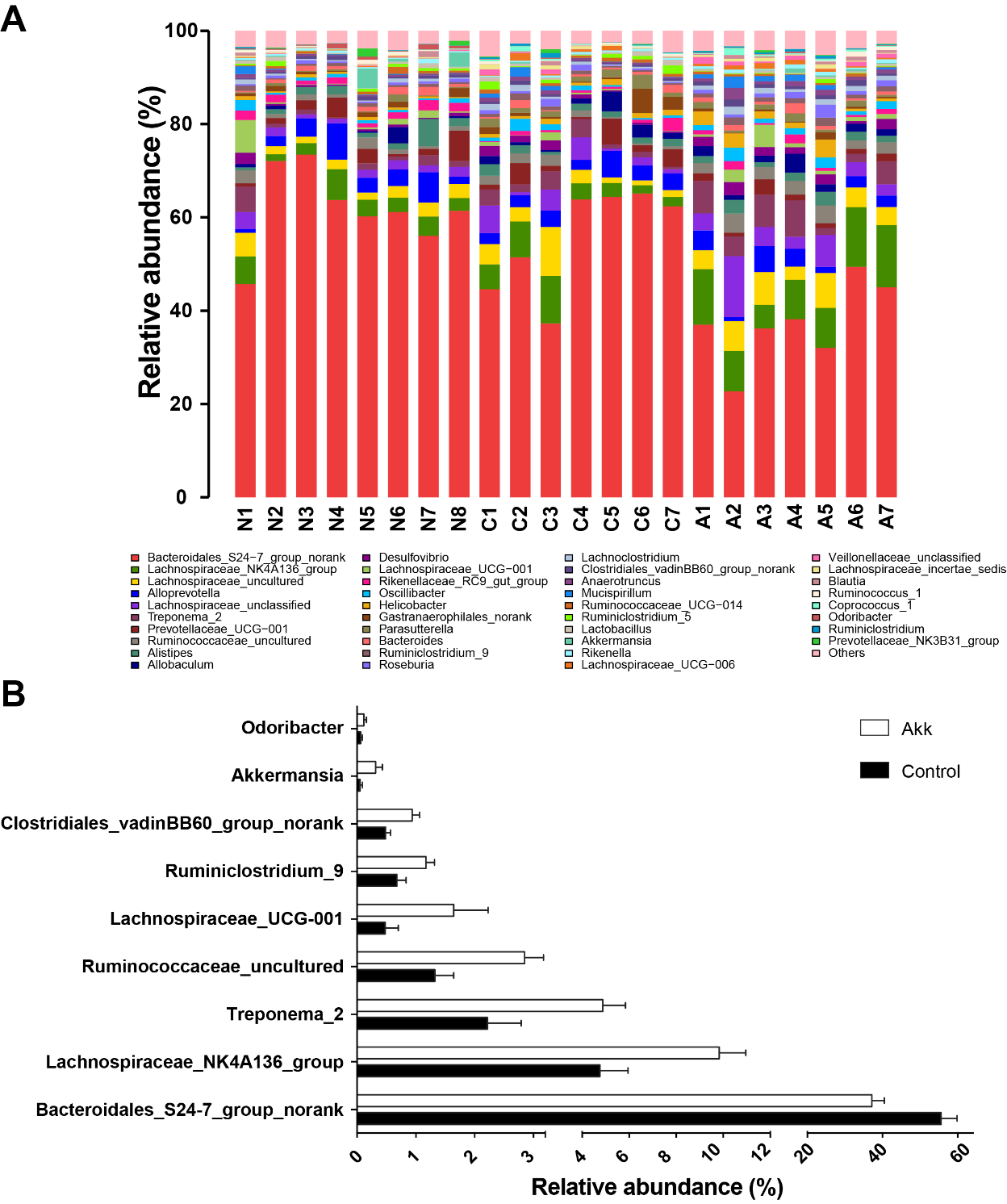


## Supplementary Tables

**Supplementary Table 1. Primers used for PCR**

| **Gene** | **Forward** | **Reverse** |
| --- | --- | --- |
| β-actin | AGTGTGACGTTGACATCCGT | GCAGCTCAGTAACAGTCCGC |
| IFN-γ | TCAAGTGGCATAGATGTGGAAGAA | TGGCTCTGCAGGATTTTCATG |
| TNF-α | AGGCACTCCCCCAAAAGAT | CAGTAGACAGAAGAGCGTGGTG |
| Bcl-2 | AGCCTGAGAGCAACCCAAT | AGCGACGAGAGAAGTCATCC |
| Fas | CTCCGAGTTTAAAGCTGAGG | TGTACTCCTTCCCTTCTGTGC |
| DR5 | TGACGGGGAAGAGGAACTGA | GGCTTTGACCATTTGGATCT |
| Tjp-1 | CTCCAGGTGCTTCTCTTGCT | TATCTTCGGGTGGCTTCACT |
| occludin | TTCCTCTGACCTTGAGTGTGG | CTCTTGCCCTTTCCTGCTTT |
| CB1 | CTGATGTTCTGGATCGGAGTC | TCTGAGGTGTGAATGATGATGC |
| CB2 | TGACAAATGACACCCAGTCTTCT | ACTGCTCAGGATCATGTACTCCTT |
| Reg3g | TTCCTGTCCTCCATGATCAAA | CATCCACCTCTGTTGGGTTC |
| Lyz1 | GCCAAGGTCTACAATCGTTGTGAGTTG | CAGTCAGCCAGCTTGACACCACG |
| Pla2g2a | AGGATTCCCCCAAGGATGCCAC | CAGCCGTTTCTGACAGGAGTTCTGG |
| DefA | GGTGATCATCAGACCCCAGCATCAGT | AAGAGACTAAAACTGAGGAGCAGC |

**
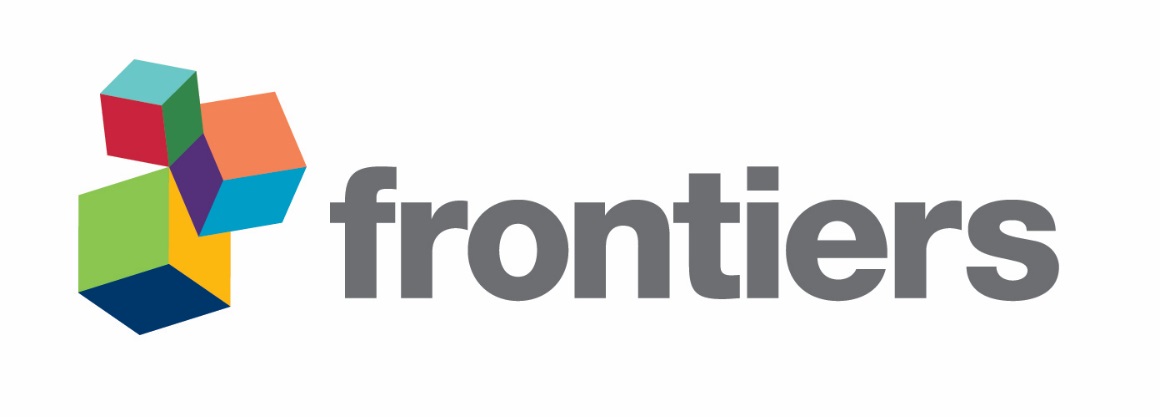
**
